# ForceGen 3D structure and conformer generation: from small lead-like molecules to macrocyclic drugs

**DOI:** 10.1007/s10822-017-0015-8

**Published:** 2017-03-13

**Authors:** Ann E. Cleves, Ajay N. Jain

**Affiliations:** 1 Helen Diller Family Comprehensive Cancer Center, University of California, San Francisco, USA; 2 Dept. of Bioengineering and Therapeutic Sciences, University of California, San Francisco, USA

## Abstract

We introduce the ForceGen method for 3D structure generation and conformer elaboration of drug-like small molecules. ForceGen is novel, avoiding use of distance geometry, molecular templates, or simulation-oriented stochastic sampling. The method is primarily driven by the molecular force field, implemented using an extension of MMFF94s and a partial charge estimator based on electronegativity-equalization. The force field is coupled to algorithms for direct sampling of realistic physical movements made by small molecules. Results are presented on a standard benchmark from the Cambridge Crystallographic Database of 480 drug-like small molecules, including full structure generation from SMILES strings. Reproduction of protein-bound crystallographic ligand poses is demonstrated on four carefully curated data sets: the ConfGen Set (667 ligands), the PINC cross-docking benchmark (1062 ligands), a large set of macrocyclic ligands (182 total with typical ring sizes of 12–23 atoms), and a commonly used benchmark for evaluating macrocycle conformer generation (30 ligands total). Results compare favorably to alternative methods, and performance on macrocyclic compounds approaches that observed on non-macrocycles while yielding a roughly 100-fold speed improvement over alternative MD-based methods with comparable performance.

## Introduction

We introduce a new method for 3D structure generation and conformational elaboration that does not rely on distance geometry, precalculated molecular templates, or stochastic sampling. Rather, it is driven by coupling intuitive physical molecular movement with the internal conformational energy computed from a molecular mechanics force field. The method is called “ForceGen” (short for Force Field Based Conformational Generation) and is implemented using an extension of MMFF94s along with a partial charge estimator based on electronegativity-equalization. Here, we report the details of the method and results on four data sets that span a large variety of drug-like molecules, including comprehensive results on nearly 200 macrocyclic compounds whose bound structures have been determined crystallographically.

Both initial 3D structure generation and conformational elaboration are centrally important calculations in computer-aided drug design. For the former task, programs such as CONCORD [1], CORINA [2], and OMEGA [3] are widely used. These approaches make use of known optimal geometries of molecular fragments (often separately considered as ring systems, non-ring substituents, and linkers) that are used as templates for constructing reasonable, low-energy 3D models of small molecules. The methods also implement fallback strategies for structure generation in the case that a molecule contains a novel structure. Such approaches can be both very fast (e.g. more than 100 structures per second) and robust. However, because producing a single 3D structure of a flexible molecule is almost invariably followed by conformational elaboration, it is the latter process that is both the time and quality bottleneck.

For conformational elaboration, our approach for many years had been to do so dynamically, tightly coupled with optimization of the objective function of a docking [4, 5] or molecular similarity calculation [6, 7]. Recently, we have explored hybrid approaches that blend agnostic conformational elaboration prior to docking or similarity optimization with some degree of local refinement during the pose optimization process [8, 9]. Agnostic conformer generation (independent of any target) can offer advantages both in terms of speed and predictive accuracy, but this places a premium on the quality of the conformational ensembles.

Quality is typically measured by the fraction of cases where a conformational ensemble produces a close match by RMSD to a conformation either from small-molecule crystal structures or from protein-ligand complexes. Clearly, algorithm speed is a serious practical consideration. For small drug-like molecules, algorithms such as OMEGA can produce conformational ensembles with times of a few seconds per molecules [3]. Recently, there has been increased interest in macrocyclic ligands in terms of their tractability by computational approaches [10–13]. However, to achieve high quality ensembles for macrocycles, the computational cost can be burdensome. Typical per-ligand times of $10^3$–$10^5$ seconds of wall-clock time (real human-perceived time as opposed to nominal CPU time) for the most effective protocols [13] limits their practical applicability.

**Fig. 1:**
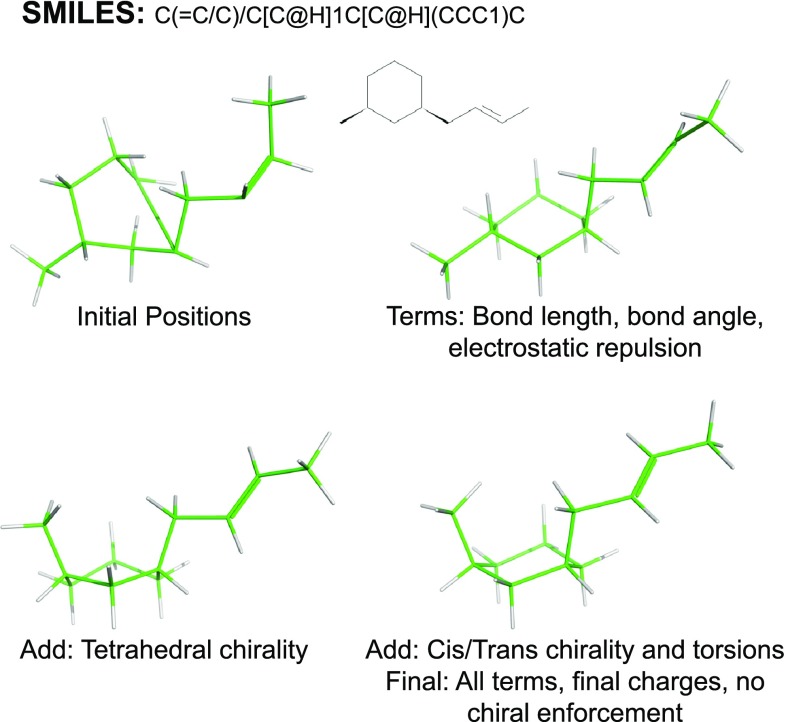
Structure generation from molecular connectivity and chirality: initial atomic coordinates are produced (*top left*); minimization with bond length, angle, and electrostatic terms (*top right*); tetrahedral chirality enforcement (*bottom left*) cis/trans double-bond enforcement (not shown); final refinement with all standard force field terms (*bottom right*)

The new methods for 3D structure generation and ring elaboration will be presented here at a high level. Specifics regarding thresholds, counts, weights, and so forth will be presented in the section on Algorithmic Details. The ForceGen method for initial 3D structure generation is depicted in Fig. 1. ForceGen builds a 3D model structure of a small molecule in the following six steps:

1. *Initial atomic positions* Given an input molecule (e.g. from a SMILES string), atom chirality and carbon-carbon double-bond configurations are noted. Then initial atomic positions are assigned recursively, with each atom being modeled as a tetrahedron irrespective of its hybridization state. Approximate bond lengths are used, and connections between different atomic tetrahedra are made to be *anti* rather than *gauche*. Molecules with rings will have a small number of poor bond lengths and geometries. Repeated iterations of initial position assignment vary the torsional angle choices and the atomic assignment within the individual tetrahedra.
2. *Rough refinement* With atomic partial charges assigned uniformly to −0.1, a Cartesian minimization is performed using the bond angle, bond length, and electrostatic terms of the force field.
3. *Tetrahedral chirality* Improper torsion terms are added to enforce the desired chirality at tetrahedral centers, and the structure is refined through minimization.
4. *Double-bond configuration* All torsional terms are turned on, and strongly weighted torsional terms are added to enforce the desired geometry across double bonds, followed by minimization.
5. *Final refinement* The special configurational enforcement terms are discarded, sensible partial charges are assigned, and all force field terms are enabled, followed by minimization.
6. *Termination and Iteration* The final refined structure is checked against the recorded chirality/bond-configurations and against an energy per atom threshold. If both tests are successful, the structure is called “correct” and the structure counts towards a minimum number of successes. If the chirality of the structure is not correct, the structure is discarded. If the structure has lower energy than the current best, the current structure replaces it. After the minimum number of successes are achieved or a maximal number of attempts are made, the procedure terminates, returning the best structure.

The example shown in Fig. 1 shows a di-substituted cyclohexane with two chiral carbons and a *trans* double-bond. The initial atomic position assignment is reasonable, except for the atoms that close the ring and those that are not supposed to be tetrahedral. Initial refinement improves the structure, but the chirality of one of the methyl substituents is incorrect, and the double-bond remains out-of-plane (torsional terms and out-of-plane terms have not been applied at this stage). The addition of terms to enforce correct tetrahedral chirality addresses that issue (lower left), as does the imposition of torsional constraints to obtain the correct configuration of the double bond (not shown). Final refinement with sensible partial charges and all normal force field terms yields a successful structure (lower right).

**Fig. 2:**
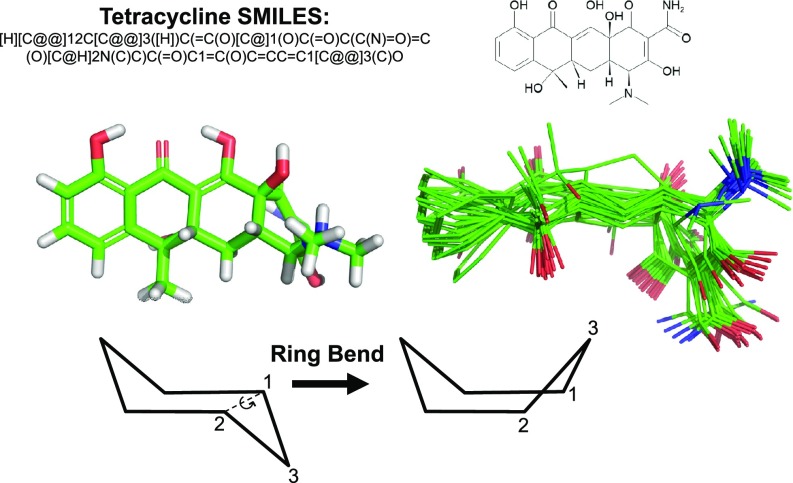
Ring bending for elaboration of ring system flexibility: initial 3D structure generation produced a reasonable conformer for tetracycline (*middle left*); ring bends are identified among atoms of a ring system according to rules, with an example for cyclohexane shown (*bottom left*); iterative application of the bends identifies new ring conformations effectively (*middle right*)

Conformer generation is similarly driven by the force field. The torsional sampling method within ForceGen is not substantially different from other approaches and will be described in Computational Methods. However, the approach to ring search is novel, both for systems composed of small flexible rings (sizes ranging from five to eight) and for macrocycles (here we have explored sizes from 9 to 32, with the bulk being from 12 to 23). Figure 2 illustrates the search method for ring systems composed of multiple small flexible rings as seen in tetracycline. The central concept is the “bend.” Such bending replicates the intuitive physical manipulation of a plastic organic chemistry model of cyclohexane to produce chair, twist-boat, and boat conformations. The procedure has five steps:

1. *Identify ring systems* Given a single reasonable 3D conformer for a molecule, ring systems are identified where all bonds between atoms of the ring system are part of rings of size three to eight.
2. *Identify ring bends* For any pair of atoms within a ring system, it will be used as a ring bend if the following three conditions hold:
  - *Not-connected* Each ring bend pair must not be directly bonded.
  - *Non-planarity* At least one atom of a ring bend pair must be part of a non-planar ring.
  - *Bridged or fused rings* The pair must not cross a bridged ring atom or a ring fusion.
3. *Identify LHS and RHS sides for bends* For each ring bend, we identify the “sides” of the bend and arbitrarily call the smaller of the two the right-hand-side. The atoms of the ring system form the RHS and LHS sides, and their pendant substituents are noted.
4. *Iterate over bends* For each ring bend, we will do the following:
  - *Make a bend* Centroid locations are computed for the LHS and RHS ring system atoms. The torsion angle is computed using the RHS centroid, the ring bend atom pair as the axis, and the LHS centroid as the last position. A rotation around the axis is made for the RHS atoms and their pendant groups such that the ring is bent opposite to its existing configuration (see Fig. 2). Neither the LHS atoms/substituents or the axis atoms/substituents are moved.
  - *Relax the bend* The atoms of the ring system are “pinned” using a quadratic positional penalty and the conformer is minimized.
  - *Finalize the bend* The pinned atoms are released, and the conformer is minimized again.
  - *Check quality and add to ring conformers* If the resulting conformer has not inverted any specified configurations, falls within an energy window of the current minimum, and is non-redundant based on RMSD of ring system atoms, it is added to a growing list of ring conformers.
5. *Termination and Iteration* This process iterates through all ring bends repeatedly until either no new ring conformers are found or a maximal number of rounds are completed.

For tetracycline, ring system identification yields the complete fusion of four six-membered rings (each with different saturation patterns). The repeated process of bending the ring system yields fifteen distinct ring conformers within 10.0 kcal/mol of that with the lowest energy. The procedure is general, not requiring any precomputation of large numbers of specific ring templates, and its pure physical manipulation is effective on diverse ring systems.

The focus of the current work is on generality and accuracy, not on speed optimization. In the current implementation, for small systems such as cyclohexane, ring elaboration takes on the order of a tenth of a second. For complex systems, timing varies depending on the rigidity of the system. Steroids such as testosterone take a few seconds, while flexible systems such as seen with tetracycline take a few tens of seconds. The time required depends entirely on the degree of flexibility that each ring system contains.

**Fig. 3:**
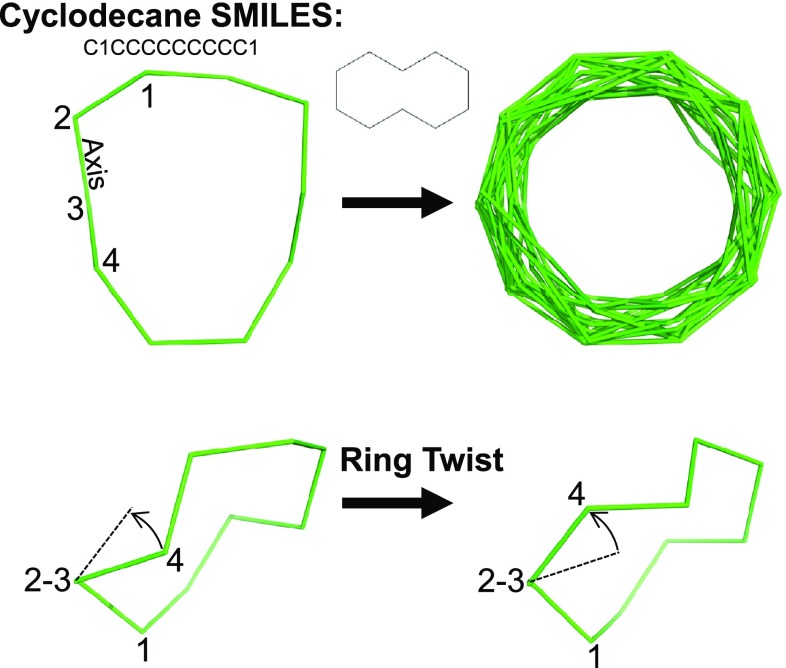
Bonds within macrocyclic rings are subjected to twists by forcing rotation around each twistable bond using positional constraints and minimization. Cyclodecane is shown as an example and yielded 28 conformations (*top-right*), the best one being within 0.01Å RMSD of the relevant small molecule crystal structure (CSD Refcode BATVOH)

For macrocyclic systems, the components that are composed of small rings are elaborated using the bending approach just described. For ring systems of size nine or larger, ForceGen makes use of an additional physical manipulation: a “twist” that is applied to force rotation around the bonds within macrocycles. Figure 3 illustrates this using cyclodecane as an example. The approach is very similar to that described for application of ring bends, and the application of macrocycle twists occurs after Step 4 in the above procedure. Any single bond within a ring whose smallest enclosing ring size is nine or greater will be twisted. Each such twist consists of the two central bonded atoms (e.g. atoms 2 and 3 in Fig. 2 along with the connected ring atoms (atoms 1 and 4). Such bonds are to be twisted, as follows:

1. *Pin the non-moving atoms* Atoms 1, 2, and 3 of the torsion are pinned with quadratic positional constraints.
2. *Rotate the other atom* A series of positions for atom 4 are identified that represent rotations around the 2–3 axis. For each of these positions, a quadratic position constraint is set, and a copy of the parent conformer is minimized subject to the pinned positions.
3. *Finalize the twists* The pins are released, and the twisted conformers are minimized.
4. *Repeat with other end* The preceding steps are redone, but with atom 1 moving instead of atom 4.
5. *Check quality and add to ring conformers* For the resulting conformers that have not inverted any specified configurations, fall within an energy window of the current minimum, and are non-redundant based on RMSD of ring system atoms, they are added to a growing list of ring conformers.

Pinning the trio of atoms during each twist holds just a part of the macrocycle in place, but it allows the remaining atoms to move so as to adapt to the forced rotation of the fourth atom. In Fig. 3, only the two closest unpinned carbon atoms to Atom 4 move significantly, with the remaining atoms reacting very little to the perturbation. This simple procedure produces 28 distinct conformations for cyclodecane in about ten seconds using default values for conformer redundancy elimination. For a much more complex macrocycle, such as vaniprevir (discussed in detail later), a potent inhibitor of the HCV NS3/4A protease (macrocycle ring size of 22 atoms with seven rotatable bonds outside the ring system), the entire conformational generation process takes a few minutes.

We present results on a standard benchmark of 480 drug-like small molecules from the Cambridge Crystallographic Database (the CSD Set) used for validation of the OMEGA method [3, 14], 667 molecules from the MacroModel ConfGen validation study [15], 1062 ligands from the PINC cross-docking benchmark with deep representation of ten pharmaceutically relevant targets (the PINC Set) [9], 182 macrocyclic ligands from protein-ligand complexes curated from the PDB (the Macrocycle Set), and 30 macrocyclic ligands that form a commonly used benchmark originally reported by Chen and Foloppe (the Foloppe Set) [11].

The focus of this work has been on conformational ensemble quality for pharmaceutically relevant small molecules, and there are a number of opportunities for speed optimization without altering the quality of the results obtained by the method. Structure generation and conformer elaboration for the ForceGen method each require seconds per molecule for non-macrocyclic drug-like ligands. However, for macrocyclic ligands, owing to the novel and *direct* method for ring twisting, conformation generation requires on the order of $10^1$–$10^3$ seconds for typical examples.

Direct comparisons with other methods were possible for the CSD, ConfGen, and Foloppe Sets. For the CSD Set, ForceGen performance was statistically significantly better than that reported for the OMEGA method [3], though the ForceGen approach may be somewhat slower (by a factor of 2–3). For the ConfGen Set, ForceGen was statistically significantly better than ConfGen, and it was also substantially faster at equivalent levels of performance (by several fold). For the Foloppe Set, ForceGen’s performance was statistically indistinguishable from the performance of the best-reported results, but it is roughly 100-fold faster. On a single CPU core, with a single-threaded implementation, the average conformational elaboration time for ForceGen was 300 wall-clock seconds, with competing methods such as Low Mode MD requiring thousands or tens of thousands of seconds on single processors.

ForceGen is a general method whose performance represents a significant advance over existing 3D structure and conformer generation approaches, particularly on macrocyclic molecules. ForceGen is implemented within the Tools module of the Surflex Platform, Version 4.0.

**Table 1:** Summary of molecular datasets and their relative complexity

Set name	Description	N	N heavy atoms	Rot. bonds	N macrocycles	Macro. size
CSD set [3]	Lead-like	480	$23.0 \pm 3.9$	$4.7 \pm 1.5$	0	0
ConfGen set [15]	Drug-like (diverse targets)	667	$25.7 \pm 8.2$	$6.1 \pm 3.4$	0	0
PINC set [9]	Drug-like (large)	1062	$29.3 \pm 10.8$	$7.6 \pm 5.2$	22	$15.9 \pm 2.2$
Macrocycle set	Diverse macrocycles	182	$40.0 \pm 11.1$	$6.7 \pm 4.5$	182	$16.7 \pm 4.3$
Foloppe set [11]	Diverse macrocycles	30	$39.6 \pm 15.6$	$6.2 \pm 5.1$	30	$18.2 \pm 8.3$

## Data, methods, and computational protocols

Where possible, data have been collected that allow for fair and direct comparisons between the methods reported here and widely used alternatives. This is challenging for three reasons: (1) some high-quality data sources prohibit redistribution of molecular structural data, necessitating re-acquisition; (2) many methodological developers and evaluators choose to provide only PDB and ligand HET codes, or only PDB codes with no indication of ligand identity, necessitating inferences as to ligand bond orders, tautomer states, formal charges, and even which ligand might be meant; and (3) molecular file format and conversion utilities may introduce noise into the data, most commonly by producing incorrect annotations of chiral atoms and configurations of double bonds. Every effort has been made here to ensure that the curated data fairly represents the structural data underpinning other published reports, and great care has been taken to remove *all* memory of 3D coordinates prior to generating initial 3D structural models and proceeding with conformational elaboration.

### Molecular data sets

The results in this work were derived from the data summarized in Table 1. The CSD Set was provided as CCDC Reference Codes in the primary validation study of OMEGA from Hawkins et al. [3]. These compounds were downloaded directly from the Cambridge Structural Database [14] as SYBYL mol2 files; the largest connected molecular graph was detected (the first one if multiple of maximal size existed) and taken to be the structure of interest; and protons were added automatically as needed (few compounds had full explicit hydrogen atoms). Because the CSD Set contained explicit bond order information, fidelity of results to other reports is expected to be high.

The remaining four data sets were curated directly from the RCSB PDB, using an automated process. Given a PDB code and a specific HET code, the procedure is as follows:

1. The PDB biological assembly is downloaded using wget.
2. The Surflex-tools grindpdb command is used to heuristically infer components (protein, water, cofactors, and ligands), bond orders, and protonation/tautomer states. SYBYL mol2 files are produced for all components.
3. Quality measurements are calculated:
  - *Ligand strain by movement* A ligand is minimized under a quadratic positional constraint on its heavy atoms. It is retained if RMSD from the original coordinates does not exceed 0.55Å. The ligand is then freed from positional restraint.
  - *Ligand strain by energy* The ligand’s pose is optimized in Cartesian space with both the internal force field and the Surflex-Dock scoring function. Its internal energy is calculated and it is then minimized outside of the protein. If the difference between the (per heavy atom) optimal pose and the local minimum does not exceed 0.50 kcal/mol/atom, it is retained.
  - *Structure quality by movement* If the RMSD between the experimental coordinates and the optimal scoring pose from local optimization is less than 1.25Å, the ligand is retained.
  - *Structure quality by ligand efficiency* The optimal docking score (nominally in units of pK$_d$) divided by the number heavy atoms is at least 0.10 pK$_d$/atom, the ligand is retained.
  - *Structure match to alternate curation* Graph matching is done between the final ligand and the corresponding SMILES-based molecular structure (and tautomeric variants) from the RCSB Ligand Expo. If there is a match, the ligand is retained.
4. In a few cases where a ligand from a reported benchmark failed this process, manual adjustment of bond orders was done (this was needed for several ligands from the Foloppe Set).

For the PINC dataset, the original report made use of 1261 ligands. Here, with a fully automated workflow, 1062 ligands emerged passing all quality criteria (84%). In cases where ligands automatically parsed from PDB coordinates fail to match the curated SMILES structures, one cannot assume that either structure is correct. In such cases, we have observed: that the grindpdb procedure yields a ligand structure that matches the published report; that the PDB curated SMILES (or SDF) structure matches the report; or that neither the grindpdb ligand nor the PDB curated structure matches the report. Further, in all cases where the independent structural inference from the Surflex-Tools grindpdb command agrees with the PDB curated SMILES structure, such ligand structures appear correct.

For the ConfGen set, the original publication listed 667 PDB codes (with no indication of ligand HET names) in supplementary material [15]. Using the automated procedure just described, the PDB structures were processed. After eliminating ligands that failed quality criteria, duplicate ligands from individual structures were removed. Cases where multiple ligands were still present were manually checked against the PDB monographs to identify the ligand of interest (typically this involved selecting an inhibitor rather than some type of cofactor). Cases where a single ligand was present were manually checked to ensure that the ligand was the compound under study, and cases where this was not true were discarded. This process resulted in 520 ligands from the original full set of 667 (79%). The remaining 147 ligands were manually curated in order to ensure that the correct structures were used. In all but a single case, the small molecule SDF files were used unmodified. The single case requiring adjustment was PDB Code 1QBV, where the coordinates for a single carbon of a phenyl ring were clearly wrong (they were on top of the carbon para to the correct position and the problem was fixed manually).

For the Macrocycle Set, the entire set of roughly 25,000 SMILES strings associated with different ligands in the PDB were analyzed. Those that were possible to parse, for which force field parameters were assignable, and where bonds existed for which the smallest enclosing ring size was nine or greater were identified. The PDB codes for that set were extracted, and these were subjected to the procedure described above. Those ligands with 60 or fewer heavy atoms which passed all quality tests formed a large set with substantial redundancy. For each protein structure, the surviving macrocyclic exemplar with the highest ligand efficiency was retained. In cases where a single HET code was represented in more than one protein structure, at most five examples were retained (again based on ligand efficiency). There were 3 ligands with 5 exemplars each, 1 with 4, 8 with 3, 13 with 2, and 113 represented as singletons.

Overall, there were 138 unique macrocyclic ligands. Multiple examples of ligands were included for two reasons. First, ligands from different protein structures often exhibit different conformations. Second, the algorithms we report here may be dependent on atom order: the sequence of bends and twists that are made can vary depending on atom ordering. Therefore, it seemed wise to consider the effect of differences in input even in cases where the bioactive conformations might be quite similar. We believe this to be the largest set of macrocyclic ligands curated for the purpose of assessing 3D structure and conformer generation. While there are sure to be additional macrocycles of 60 or fewer heavy atoms of high quality in the PDB, we believe that this set is both diverse and large enough to begin to tease apart statistically significant differences between the performance of different methods.

The Macrocycle Set contained 14 of the 30 molecules from the Foloppe Set, the remainder of which were curated as just described, but several ligands required careful manual correction of bond orders.

In all cases, the coordinates of non-hydrogen atoms were not changed in building the reference ligand poses. In order to assess either 3D structure generation or conformer generation, we believe that it is important to fully erase any memory of the target coordinates. Here, we have taken two approaches. For all five data sets, we used a procedure to mark tetrahedral chirality and carbon-carbon double-bond configurations and then *zeroed* all coordinates. This computational molecular construct consists only of atomic elements, bond connectivity, formal charges, and the two types of configurational notations. It contains the same information as an isomeric SMILES representation. For the CSD Set, in addition, we repeated our experiments using isomeric SMILES as input.

### Algorithmic details

There were four major additions to the Surflex Platform for the work reported here: a more sophisticated force field, a partial charge assignment method, a method for 3D structure generation, and a novel ring search method. They are detailed as follows, along with a brief description of the torsional sampling approach, which has not been substantially altered.

#### Force field: MMFF94sf

Our variant of MMFF94 and MMFF94s [16–21] is called “MMFF94sf.” The implementation began directly with MMFF94 (including analytical gradients), followed by the parameter changes introduced in MMFF94s [21] that increased the planarity of unstrained delocalized trigonal nitrogen centers. Extensive validation was conducted against the two available suites of small molecules with assigned atom types, energetic term values, and total energies.

The validation tests were done using a dielectric value of 1.0 and the partial charges given. We can technically call our implementation of MMFF94s a “partial” one, because on fewer that 3% of the molecules in the validation suite, our atom type assignments differ slightly. These differences typically occur in the treatment of nitrogen atoms where there are multiple logical assignments for the atom types, generally in aromatic or conjugated systems that also include a formally charged nitrogen. The differences were very small in terms of the locations of the minima between our implementation and a fully compliant one (hundredths of Angstroms).

Of more significance, we have further modified the force field to increase the planarity of unstrained delocalized trigonal nitrogen centers. For each out-of-plane term within MMFF94s that differed from one in MMFF94, we multiplicatively alter the force constant by a fixed value, whose default is 6.67. Changing this value to 1.0 brings the parameters back to those in MMFF94s. No adjustments were made to the torsional terms of MMFF94s.

We call our variant force field MMFF94sf to distinguish it from other variants. In all of the work reported here, we used a dielectric constant of 80.0 to match aqueous conditions. This has the beneficial effect of preventing intramolecular electrostatic interactions from dominating the energetic minima and allowing molecules to explore a wider range of conformational configurations. Partial charges were assigned as follows.

#### Partial charges: electronegativity equalization

Rather than using the bond-charge-increment scheme of MMFF94 [17], we have implemented an electronegativity equalization approach, similar to that reported by Gilson et al. [22]. Electronegativity equalization is a general method for partial charge assignment that avoids complex atom typing schemes [23–28].

The Gilson method was parameterized with 39 atom types, each associated with an electronegativity and a hardness (which quantifies an atom’s resistance to change in preferred charge). The atomic electronegativity values are modified based on local environment. Then, atomic charges are assigned by minimizing a simple function *E* that depends on the electronegativity and hardness of the atoms in the molecule. The function *E* is defined as follows, where for each atom *i*, $e_i$ is the modified electronegativity, $s_i^o$ is the hardness, and $q_i$ is the partial charge:

1 $$\begin{aligned} E&= \sum \limits _{i=1}^{n}\left( e_iq_i + \frac{1}{2}s_i^oq_i^2\right) \end{aligned}$$

Minimization of *E* is subject to two constraints. First, formal charge within local groups of atoms is preventing from bleeding outside of each group by a fixed amount. Second, the total formal charge of the molecule must be the sum of the individual partial charges. One last aspect aspect of the method was a novel approach to ensure that atomic equivalence over different resonance forms resulted in equivalent charges for equivalent atoms. The parameters of the method were chosen to reproduce *ab initio* molecular electrostatic potentials for a set of 284 molecules.

Our method differs in two key respects. First, to address the issue of symmetry across different forms of a molecule, instead of enumerating resonance forms, we make use of a general graph matching algorithm that identifies atom-atom equivalencies. This is done through straightforward topological comparison of a molecule to itself (the same approach is used to identify chiral atoms). The partial charges of any atom sets that are identical to one another have their initial charges replaced by the mean across the symmetry group. For example, in propane, the methyl groups on the ends form groups of six identical hydrogen atoms and two identical carbon atoms, with the middle carbon being unique but having two identical hydrogen atoms.

Second, our method differs in the manner in which the constraints on the total formal charge and on local formal charge containment are enforced. The Gilson method makes use of Lagrangian multipliers over $3^N$ different conditions, where *N* is the total number of charge groups, and the charge distribution with the lowest value of *E* is taken. Instead, we perform a series of minimizations of *E* using Powell’s method [29], with a quadratic penalty on the magnitude of violation of the total charge and local charge constraints.

The quadratic penalty is weighted by a factor of 2 in the first iteration (e.g. if the formal charge of a molecule is 0.0 and the total partial charge is 2.5, then the penalty applied to *E* is 12.5). Beginning with the optimized partial charges of each successive minimization, the penalty on deviations is doubled, and the process is iterated until the penalty exceeds $10^8$. Using this method, deviations for either total charge or local charge are smaller than $10^{-6}$.

For the current work, parameterization followed that of Gilson et al. [22], but additional refinement will be the subject of future work. In particular, we plan to make use of MMFF94sf atom types, which form a more diverse and complete description of chemical behavior, on a much larger set of molecules for parameterization. Also, rather than using a hard boundary on local charge bleed, it is appealing to contemplate a softer penalty and see whether the fit to high-quality *ab initio* charges can be improved. Note that because we have made use of a high dielectric constant here, the effects of small differences in computed partial charges are expected to make little impact on the character and quality of the conformational ensembles produced by ForceGen.

#### 3D structure generation

The Introduction described the overall algorithm for 3D structure generation. Important details in the implementation are as follows:

1. *Bond lengths* For initial atomic position assignment, bonds between hydrogen and any other atom are given the standard alkane C–H bond length from MMFF94. All other bonds are assigned the standard alkane C–C bond length.
2. *Minimization* The process of structure refinement requires repeated minimization calculations, each potentially turning off or on various classes of terms in the forcefield. All of the minimizations are done in Cartesian space, using the quasi-Newton Broyden–Fletcher–Goldfarb–Shanno algorithm (BFGS). In the initial refinement of the structure (Steps 1–5), the termination conditions are gradient $\le 0.1$, atom position change $\le 0.001$, and energy change $\le 0.1$. For final refinement, these threshold values become, respectively, $10^{-3}$, $10^{-5}$, and $10^{-5}$.
3. *Iteration* Refined molecular structures whose final energy is $\le 7.0$ kcal/mol/atom and whose specified chirality and double-bond configurations are correct is called a success. The process of initial atomic position assignment and refinement is repeated until either six successes occur *or* until a maximum number of tries is exceeded, defined by default to be five times the number of atoms in the molecule.

In the event that the procedure fails to find any successful structures (as defined above), if a structure has been produced that matches the specified configurations of chiral centers and double bonds, that structure is returned, despite having relatively high energy. Note that for certain highly-strained molecules (e.g. cubane), the global minimum conformer has high per atom energy. In the event that no structure is found that matched the given specification, no solution is returned.

Note that it is possible, for example, to construct a SMILES string where the specified chirality is physically impossible to obtain, and this sometimes happens in error. In extensive testing, the procedure very rarely fails to find a reasonable 3D structure for a well-formed input molecule that has defined atom types within the force field. Approximately 95% of final structures have energies of 2.0 kcal/mol/atom or less, with 99.5% having enrgies of 2.5 kcal/mol/atom or less. Typical times for structure generation on the molecules under study here ranged from 1–3 s, including the atomic partial charge calculation.

With respect to protonation, the default approach is to make use of the specified formal charges from the input representation and to fill out the standard valences of the heavy atoms with hydrogen atoms. Optionally, a heuristic method may be employed to infer charges such that addition of hydrogen atoms will yield structures likely to be physiologically relevant (e.g. with carboxylic acids deprotonated).

#### Ring search

The Introduction described the overall algorithm for ring search, involving manipulation of ring systems through a series of bends and twists. Important details in the implementation are as follows (for the standard search protocol):

1. *Ring redundancy* For non-macrocyclic ring systems, ring redundancy depends on ring system size. For systems of fewer than ten atoms, the RMSD threshold is 0.1Å. For larger systems but with fewer than 35 atoms, the threshold is 0.2Å. For still larger systems, the threshold is 0.3Å. For macrocyclic systems, the entire molecule is considered to be part of the nominal ring system because pendant groups often have a strong influence on ring geometry. For these, the RMSD threshold is 0.5Å. As new conformations for ring systems are produced, they are compared to existing ones in a pool that is initialized with the original ring system configuration. In cases where a new variant is non-redundant and falls within a energy window of the current minimum, it is added to the pool. In cases, where one is redundant of an existing variant, the one with lower energy is retained as part of the pool.
2. *Atom pinning* In the process of ring bends and twists, certain atoms are held in place during minimization calculations by means of a quadratic penalty. For bends, after the physical bend is made, atoms of the ring system are pinned to their positions with a force of 100.0 kcal/mol per Å${^2}$. So, a deviation of 0.1Å from the pinned position produces a penalty of 1.0 kcal/mol. The pinning process allows the remainder of the molecule to adapt to the new ring conformation. Without this step, ring bends will often revert to their original position due to the influence of pendant atoms. For the twists in macrocyclic systems, the penalty is the same for all four atoms involved in the twist. However, the three atoms that are fixed are allowed the freedom to wiggle by 0.1Å without incurring a penalty (this results in a square-bottomed quadratic penalty). Allowing some motion for these atoms helps in accommodating the extensive physical ring-closure constraints while rotating the fourth atom around the bond axis of the twist. As in the process of structure generation, minimization is done in Cartesian space.
3. *Bends* As described in the Introduction, bends are made in flexible ring systems by identifying appropriate pairs of ring atoms whose axis will be used to perform a bend. The bend amount is determined by considering the torsion angle between the centroids of the LHS and RHS sides. The bend that is made is symmetric, so if the angle of deflection from a plane through the LHS for the RHS side is 20 degrees, then the bend will result in 20 degrees in the other direction. After each bend, a minimization is carried out with the ring system atoms pinned using the more lenient of the termination cutoffs above. Then the pins are released and a minimization is carried out with the more stringent cutoffs. This process reliably produces sensible variations of ring systems composed of small rings while preventing reversion to the original ring conformation unless the new one is inappropriate.
4. *Twists* The process of twisting is similar to that of bending, but for each twist, multiple rotations are employed in increments of 60, 120, 180, and 240 degrees. For each rotation of the fourth atom, the other three atoms are pinned in their parent conformer’s position. The fourth is pinned in the desired new positions sequentially. Minimization with a lenient cutoff is carried out initially for each such position, pins are released, and minimization with more stringent cutoffs is done. This process produces low-energy variations of macrocyclic systems that overcome high-energy barriers in a direct manner. Stochastic approaches are often stymied by these barriers, instead relying, fundamentally, on the luck of the draw to identify new ring system variations across high-energy barriers.
5. *Bounds on ring pool variants* Limits are respected both regarding the number and maximal energy of ring variants in the pool. The number depends on the search mode. For standard search. the limit for non-macrocyclic systems is 20 and for macrocyclic systems is 36. The energetic limit on ring variants in the pool, measured from the current minimum-energy ring variant, is double the overall energy window for the whole molecule. By default, the overall window is 10.0 kcal/mol, so ring variations may include conformations up to 20.0 kcal/mol higher than the particular minimum in a pool that is to be augmented. At the end of each round of twists and bends, the ring variant pool is pruned to ensure that the number and energy constraints are maintained.
6. *Iteration* When any particular ring variant is elaborated through bends and twists, it is marked as done. If it is replaced by a lower-energy alternative within the ring redundancy threshold, the new ring variation must be re-elaborated. The overall process of ring elaboration ends when no ring variants have been elaborated *or* when five rounds of ring elaboration have occurred.

This procedure has not been optimized heavily, except for small systems such as cyclohexane. Numerous opportunities exist for speed improvements and for different types of physical manipulations of ring systems.

### Torsional elaboration

The procedure for torsional elaboration has not changed appreciably since the introduction of the Surflex Docking method [4].

Briefly, the non-ring bonds within a molecule that require sampling are identified and assigned types that, for example, differentiate sp3–sp3 linkages from sp3–sp2. Different types of bonds are assigned different sampling levels (e.g. sp3–sp3 are assigned three total rotations including the existing one and sp2–sp3 bonds are assigned 6). The limits on energy and numbers of conformers in the following refer to the standard search mode. The following describes the search for a single ring variant, which is used to initialize the conformer pool. Multiple ring variants can be searched serially or as a pool. When done as a pool, the pool sizes are increased accordingly.

Groups of such bonds are made such that they form molecular fragments where each such fragment contains bonds of a single group. The groups are limited such that exhaustive sampling will not exceed 200 variants. The combination of bond rotations required to exhaustively sample the bonds within the group is applied to each conformer in the current pool, and these variants are collected and then added to the pool. From these, the most diverse subset is chosen up to 400 (the maximal pool size is larger than the maximal variant sampling because of iteration). The process is repeated through the different bond groups, with iterative steps of pool expansion and then selection based on diversity.

At this point, the conformers in the pool are rapidly relaxed using internal coordinates to minimize energy. Redundant conformers and those with excessively high energy are discarded. These are then minimized in Cartesian space using lenient cutoffs. Those that meet an energy threshold of 20.0 kcal/mol above the current minimum are then minimized using stringent cutoffs. All conformers with energy greater than 10.0 kcal/mol above the discovered minimum are discarded. If more conformers exist than desired (200 in standard mode), the most diverse of the set are chosen and returned.

In cases where the nominal calculation of the total number of conformers exceeds $10^6$ a different approach is taken. Exhaustive sampling across the identified rotatable bonds can be done in a specific order that is analogous to counting, where each digit in the count is the index of the rotation of the rotatable bond in question. For highly flexible molecules, a modulus *M* is selected such that sampling every $M^{th}$ of the sequence of conformers will produce five times the number of desired final conformations. These are then put through the same process as just described to yield a diverse pool of low energy conformational variants.

### Computational procedures and statistical analysis

The results reported here were generated using Surflex-Tools version 4.057. The bulk of the results were generated through zeroed-coordinate conformer randomization in standard search mode, as follows (shown for the PINC Set): 
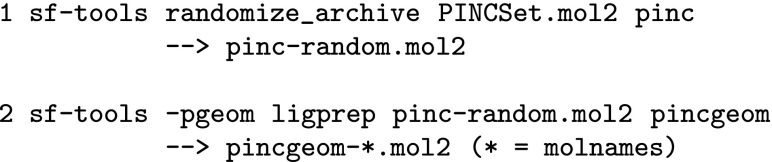
 RMS deviations were done for each resulting conformer pool by identifying all molecular symmetries, then applying the rigid body alignment transform to each conformer so as to minimize the RMSD against the crystallographic one under all identified symmetric self matches. The minimum such RMSD value (for non-hydrogen atoms) is the value reported for each ligand. RMSD of heavy atoms corrected for molecular automorphism is standard in evaluations of docking calculations and for conformer generation.

 An alternative control used for the CSD Set involved beginning the process from SMILES representations of the molecules, as follows: 
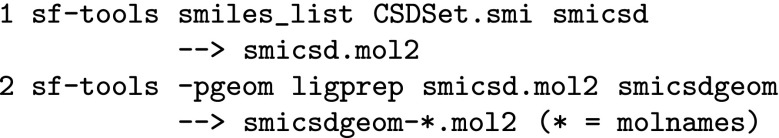
 Here, SMILES strings were generated from the original SYBYL mol2 archive file using the BIOVIA Discovery Studio Viewer Version 4.0. Note that this procedure yielded structures with very different atom orderings than in the original ligand structures, providing data to assess whether the nominal order dependencies within the ForceGen algorithms produce significant variations in conformer quality.

Standard search depth is specified by the *-pgeom* argument. This selects ring search (maximum ring variants 20 or macrocyclic variants 36) and produces a maximum of 200 conformers per ligand, with a limit of 10.0 kcal/mol for the highest energy conformer above that with the minimum energy. The thorough protocol is specified by *-pquant*, which increases the allowable ring variations for macrocycles to 72 and the maximum number of conformations to 1000 (ring redundancy for macrocycles is also decreased to 0.3Å).

The screening protocol is specified by *-pscreen*, which disables macrocycle searching, decreases ring variations to 4, decreases the energy window to 5.0 kcal/mol, and limits the number of conformations to 20 for molecules with 5 or fewer rotatable bonds, 60 for 6–11 rotatable bonds, and 200 for 12 or greater. The fast search protocol (*-pfast*) is similar to the screening one except that it disables ring search altogether, using the ring configurations in the given conformer and limits all molecules to 20 conformers regardless of the number of rotatable bonds that each contain.

Under all search protocols, conformers are eliminated whose RMSD is 0.25Å or less, though the pressure to identify maximally diverse conformer pools generally prevents this level of redundancy from occurring. Numerous user-settable parameters allow control over the procedures beyond the four basic protocol choices. However, in our experience, these four protocols cover nearly all needs.

Where possible, we have made direct comparisons between the methods introduced here and other widely used methods. This involves comparing success rates across different thresholds of RMSD for generated conformers against the experimental structures of the ligands. We favor providing full cumulative histograms of such data, which allows for detailed inspections of performance across all thresholds of interest, and it also allows for comparison of distributions using the Kolmogorov Smirnov (KS) statistical test. The KS test finds the maximal gap between two cumulative histograms, and, given the numbers of data points for each cumulative histogram, it is possible to relate the magnitude of that gap to the probability that a gap of such magnitude would be observed by chance. Note that this test is non-parametric, requiring no assumptions about the characteristics of the underlying distributions.

Technically the test tells only whether the two distributions are different, not whether one is better than the other. However, with non-perverse distributions, it is easy to see by inspection which of two cumulative histograms is better than the other given that the two are different. Here, comparisons between methods or method variations are made with data set sizes of 30, 182, 480, 667, and 1062. Respectively, the critical values for maximal percentage point differences at $p = 0.05$ are: 35.1, 14.3, 8.8, 7.4, and 5.9. So, on 480 data points, if the largest difference between Method X and Method Y is 8.8 percentage points, then Method X is likely to be statistically significantly better than Method Y at the $p = 0.05$ level. In the results that follow, an indication that a difference between methods or method variations being statistically significant refers to an approximate KS test, as just described, unless otherwise noted. Care has been taken to ensure that misleading conclusions are not suggested by clever selection of statistics or thresholds.

Additional details about the data set and computational procedures are available at www.jainlab.org. Details about obtaining the Surflex software are available at www.biopharmics.com.

**Fig. 4:**
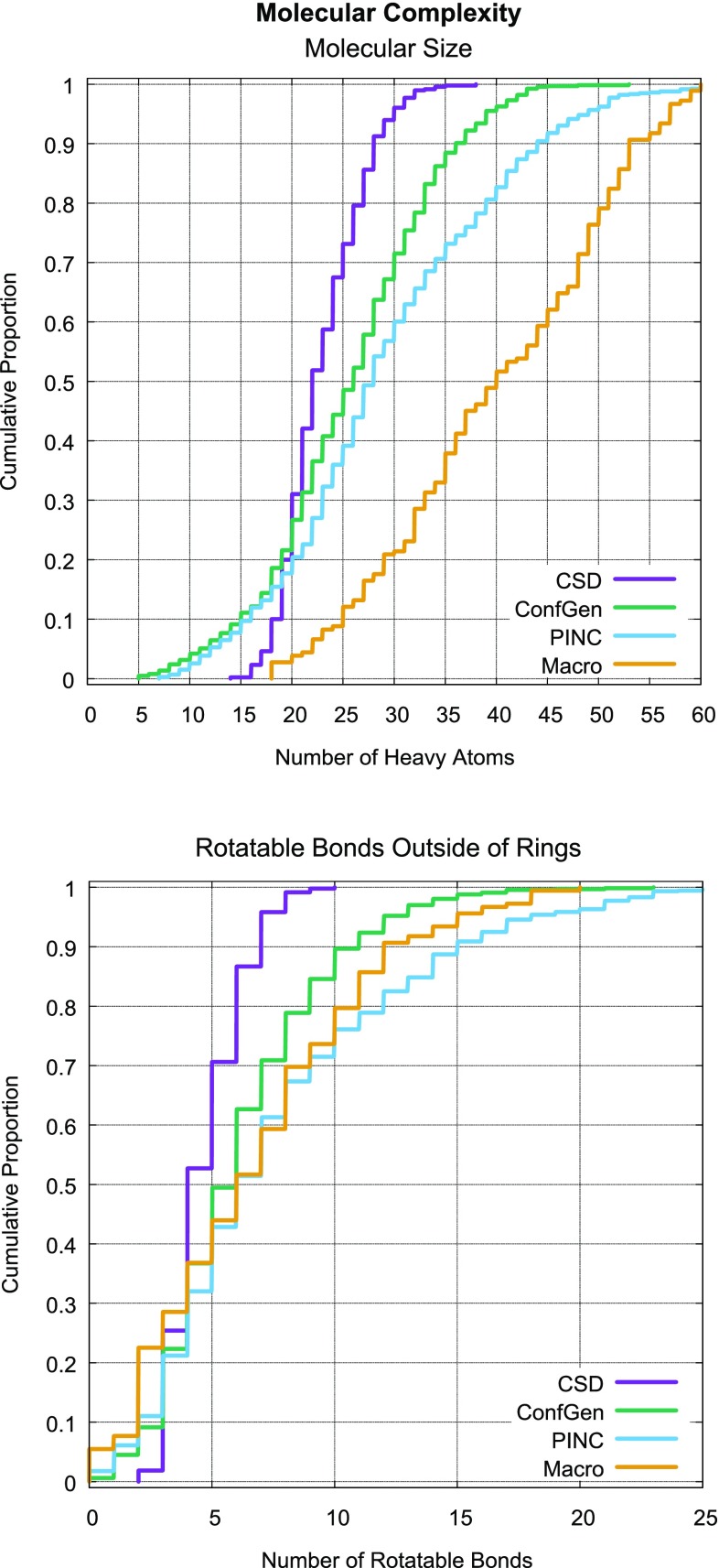
Comparison of molecular complexity for the primary data sets

## Results and discussion

Five data sets were studied in the course of this work, each addressing a different aspect of 3D structure generation and conformer generation and comparison to other approaches. Results will be presented in order of least to greatest challenge.

### Data set complexity

Figure 4 depicts the gross molecular complexity of the primary data sets. The CSD Set [3], used for OMEGA validation, contains, by far, the most simple ligand structures, both in terms of molecular size and typical flexibility. The ConfGen Set [15], used for validation of the Schrödinger conformer generation engine, is more representative of drug-like molecules. It includes a broader range of sizes, overall flexibility, presence of aliphatic rings, and it also covers a broad variety of protein targets.

The two ligand sets curated for this work are yet more complex. The PINC Set has a large number of ligands (1062) for a small number of targets (10), and it includes a higher fraction of larger and more flexible molecules than the CSD or ConfGen Sets. The Macrocycle Set of 182 ligands contains the most complex examples in all respects. In addition to each molecule containing at least one macrocycle, the molecules are larger and contain a similar distribution of rotatable bonds *outside* of ring systems as the PINC Set. The set of 30 macrocycles from Chen and Foloppe [11] were nearly identical in molecular complexity to the much larger Macrocycle Set (data not shown).

**Fig. 5:**
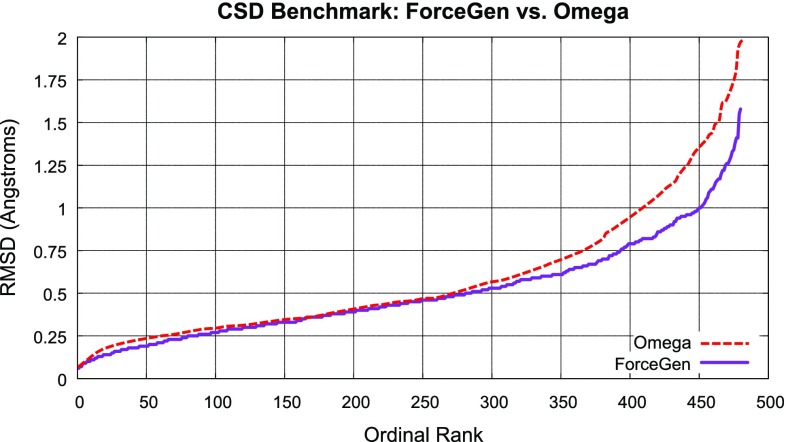
ForceGen and OMEGA results on conformer generation for the CSD Set (ordinal ranks for each molecule come from the sorted RMSD results from low to high, with the best result being 1 and the worst being 480)

### CSD set: comparison to OMEGA

Hawkins et al. published a comprehensive description of OMEGA and its application to conformer generation [3], focusing on two ligand sets, one from the CSD (480 compounds) and one from the PDB (197 compounds). Here, because the remainder of the work focuses on many hundreds of challenging protein-bound PDB ligands, we have applied ForceGen to the CSD collection, which facilitates direct comparisons.

Figure 5 shows the performance of ForceGen and OMEGA, each using a standard protocol with a maximum of 200 conformers. Among the best results (low ordinal positions at left), there is a very slight advantage to ForceGen, but at the more challenging end of the spectrum, the difference becomes more pronounced. At the 1.0Å RMSD cutoff, OMEGA yielded a success rate of 84.5%, with ForceGen yielding 94.5% success. The difference is statistically significant ($p<$ 0.025 by the KS test). Median time for conformer search was 2.3 seconds per molecule for ForceGen (wall-clock time, single processor, single-threaded, using an Intel Core i7-4770 CPU at 3.40GHz released in 2013), which is likely to be slightly slower than OMEGA.

**Fig. 6:**
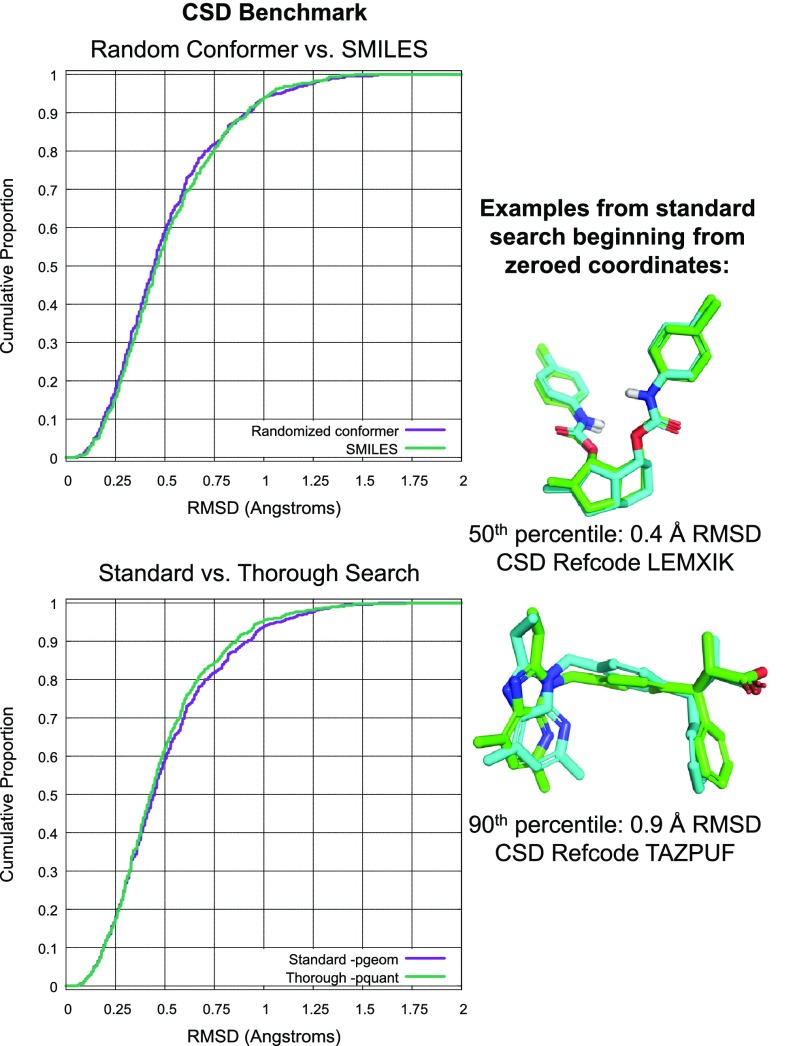
ForceGen comparison of SMILES versus zero-coordinate protocols (*top*) and standard (-pgeom) compared with thorough (-pquant) search (*bottom*) for the CSD Set

Ebejer et al. [30] compared multiple freely available methods for conformer generation to OMEGA using the CSD set. The best performing method, RDKit, yielded 90.4% success on the CSD set at the 1.0Å success threshold, better than OMEGA but worse than ForceGen.

**Table 2:** Results on the ConfGen Set, with data and ConfGen results curated from Watts et al. [15]

Program and Mode	% $\le 0.5$	% $\le 1.0$	% $\le 1.5$	% $\le 2.0$	Mean N conf.	Mean time
ForceGen fast (-pfast)	30	63	84	95	14.7	0.88
ForceGen screen (-pscreen)	35	74	91	99	39.4	8.8
ForceGen standard (-pgeom)	43	83	96	99.7	101.3	14.4
ForceGen thorough (-pquant)	47	90	99	100	348.6	68.4
ConfGen very fast	16	52	84	96	14.3	0.49
ConfGen fast	20	55	82	94	13.2	1.09
ConfGen intermediate	21	65	85	97	37.9	3.33
ConfGen comprehensive	35	71	90	99	146.4	8.00
ConfGen + min very fast	21	60	87	97	16.3	4.2
ConfGen + min fast	24	70	90	98	43.0	34.3
ConfGen + min intermediate	38	75	90	98	111.9	41.6
ConfGen + min comprehensive	38	76	92	99	128.2	45.8

The columns indicate the percent of the 667 molecules for which a conformer was generated within either 0.5, 1.0, 1.5, or 2.0Å RMSD of the crystallographic pose

Figure 6 shows a comparison of ForceGen performance beginning from either SMILES input (upper plot, green line) or zeroed-coordinate conformers (upper plot, purple line). The differences are neither practically nor statistically significant, and the remainder of the results will be presented using the zeroed-coordinate approach. In the bottom plot of Fig. 6, we see that for the relatively small molecules of limited flexibility within the CSD set, the difference between standard and thorough search was marginal. Shown at right is a typical case (0.5Å RMSD) on CCDC refcode LEMXIK (crystallographic pose in green and closest generated match in cyan). Note that the flexible ring system was searched dynamically as part of conformational generation. An example from the most challenging end of the spectrum was TAZPUF, where deviation from the crystallographic pose was driven by relatively small torsional minima preferences.

### Comparison to ConfGen on diverse ligands

The ConfGen Set contains a very large variety of pharmaceutically interesting ligands, spanning a diversity of protein targets. Table 2 summarizes performance results and search time of the ForceGen compared with the ConfGen method [15].

Considering similar computations (ForceGen compared with ConfGen with minimization) addresses the issue of sampling quality. Here, at each level of search depth (with each producing similar numbers of conformers), ForceGen showed a performance advantage of 5–10 points, with larger advantages at the more stringent success cutoffs. In fact, ForceGen standard performance performed even better than ConfGen’s comprehensive approach with minimization. Note, however, that the mean time for the ForceGen standard approach was 14.4 s compared to 45.8 s for the ConfGen comprehensive approach with minimization. So, ForceGen was significantly faster than ConfGen (even accounting for likely differences in hardware performance on floating-point calculations).

**Fig. 7:**
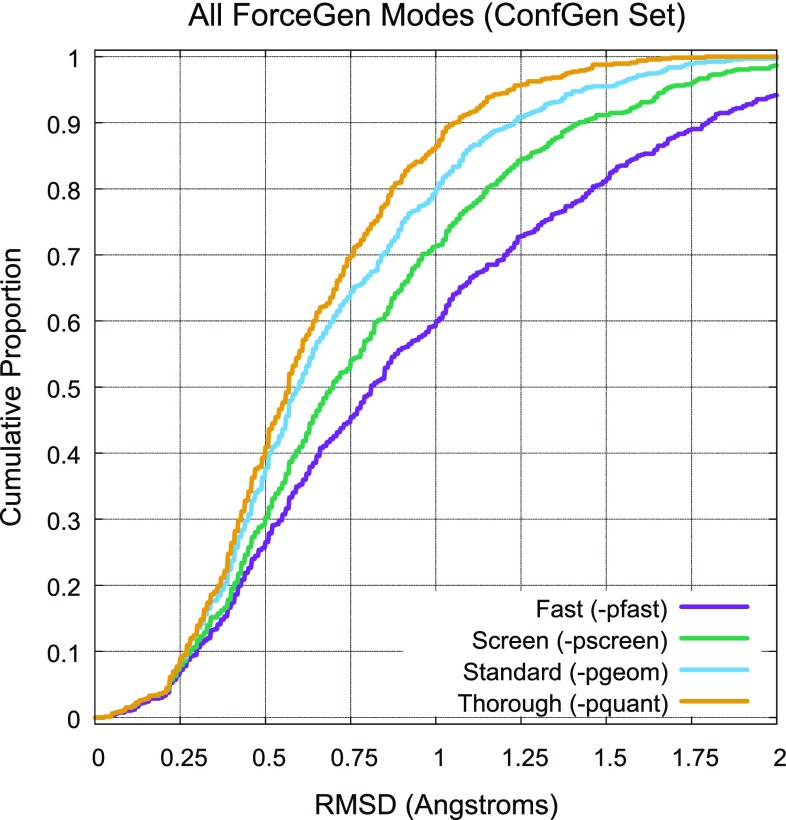
Comparison of all search modes for ForceGen on the ConfGen Set

ForceGen in its fastest mode (less than 1 s per molecule) produced results that were nearly equivalent to ConfGen’s comprehensive mode (no minimization), but ForceGen was several-fold faster. Similarly, ForceGen in screening mode produced slightly better results than ConfGen’s intermediate mode (with minimization), but was approximately 4-fold faster. ForceGen in standard mode produced better results than all variants of ConfGen. In thorough search mode, performance gains for ForceGen were roughly 5 percentage points at the 1.5Å threshold. Figure 7 shows the cumulative histograms of performance for all four of ForceGen’s search modes.

Where the maximal success percentage difference between the different methods in Table 2 is seven percentage points or greater, the difference is statistically significant ($p < 0.05$ by KS test). So, ForceGen’s performance is statistically improved by increasing search thoroughness between each of the four modes. ForceGen’s standard search mode was significantly better than all of the ConfGen variations based on the performance gap at the success threshold of 1.0Å ($p < 0.05$).

Note that performance difference for ForceGen between the CSD set and the ConfGen Set was relatively small (roughly 10 percentage points at 1.0Å RMSD). However, Ebejer et al. [30] observed a very substantial performance drop (20 percentage points at 1.0Å RMSD) for the best reported method, RDKit, when moving from CSD ligands to the PDB ligands from the OMEGA benchmark [3] despite having complexity similar to the ConfGen Set.

**Fig. 8:**
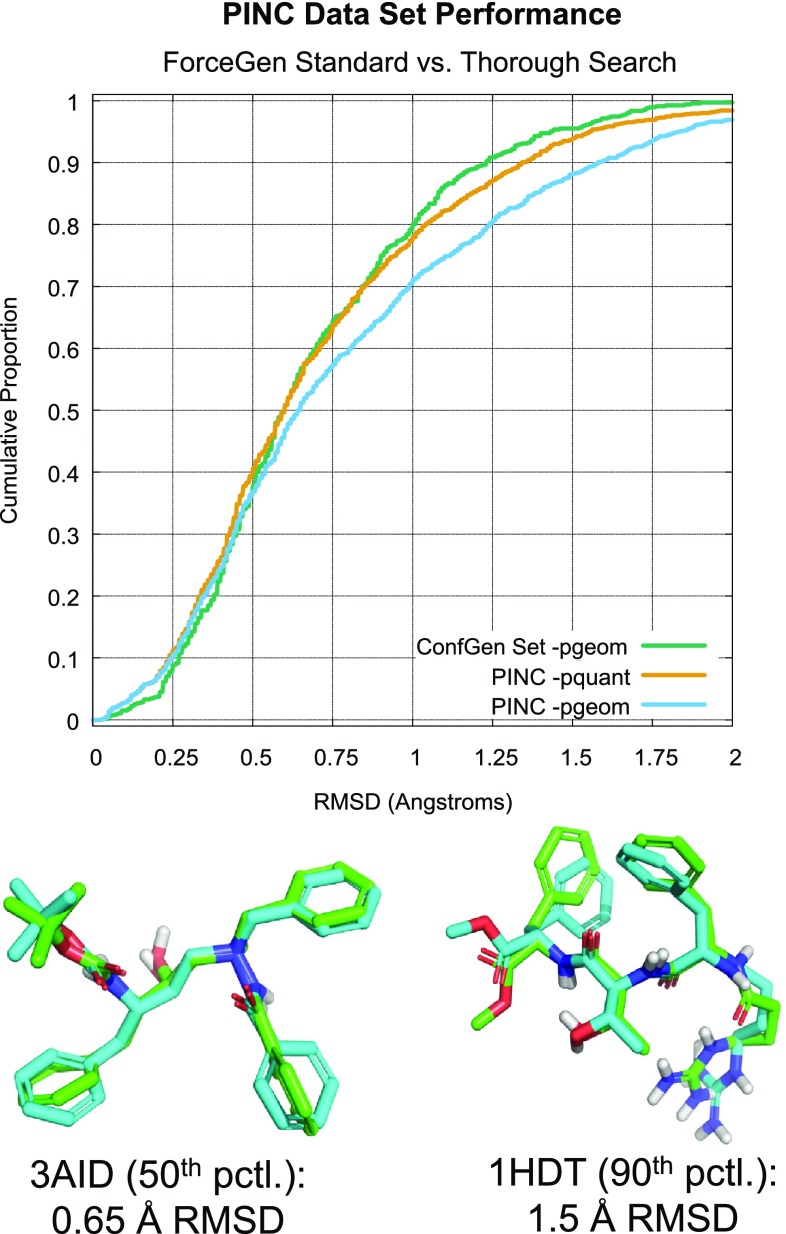
Performance on the PINC Set: both standard and thorough search

### PINC: ten targets with many diverse ligands

The structural motifs within the PINC Set cover a broad range of complex non-planar ring systems and heterocycles. These ten targets represent a diverse set of protein types: a tyrosine phosphatase (PTP1b), two aspartyl proteases (BACE1 and HIV-PR), a mitogen-activated protein kinase, (MAPK14), a serine-threonine kinase (CDK2), a serine protease (thrombin), a ligand-modulated transcription factor (PPAR$\gamma$), a metal-dependent dehydratase (CA-II), a heat-shock protein (HSP90), and a transcriptase (HIV-RT). All are either targets of existing drugs or have been actively pursued as drug targets.

**Fig. 9:**
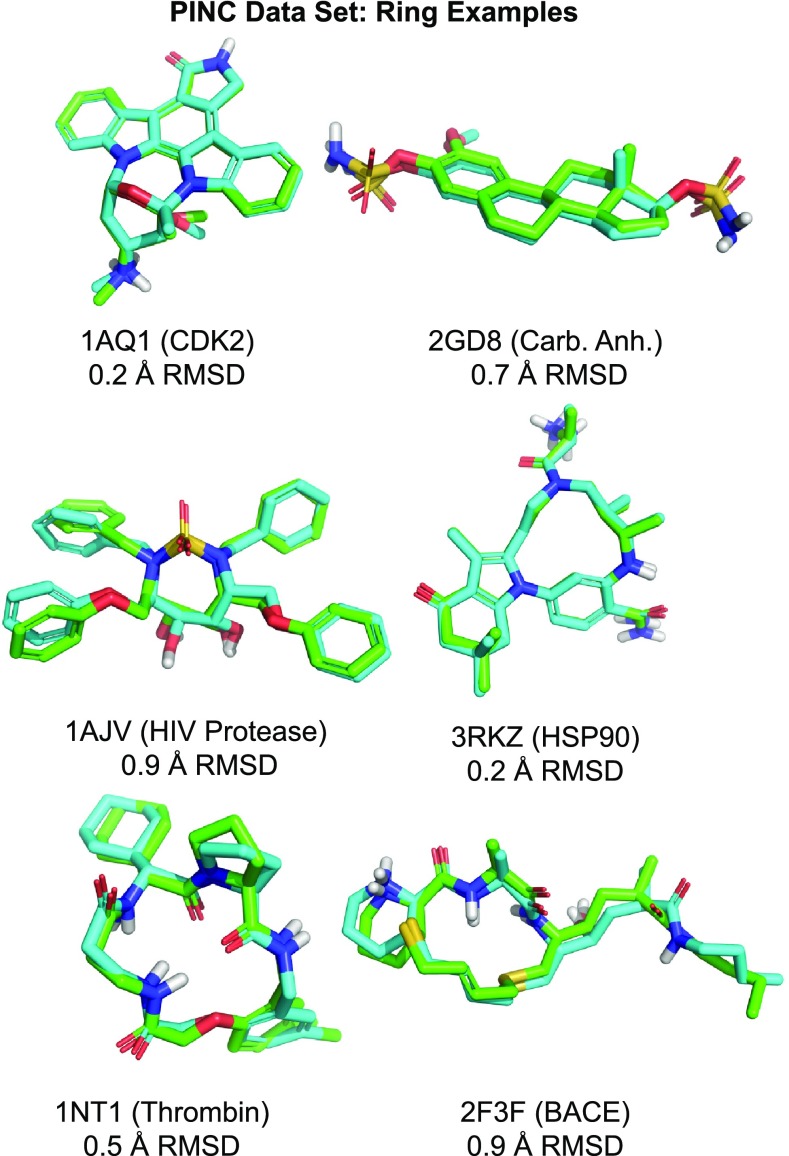
Typical examples of ForceGen performance on complex ring systems in the PINC Set

Owing to the greater molecular complexity of the PINC set, ForceGen standard search results yielded success rates of roughly 10 percentage points lower than for the ConfGen Set (see Fig. 8). However, thorough search yielded results for the PINC set that nearly equalled standard search for the ConfGen Set. The sampling methodology was able to effectively uncover bioactive poses, but the required search depth was greater.

Even with standard search, and given the presence of very large and flexible ligands in the PINC Set (including 22 macrocycles), over 95% of cases yielded a conformer within 2.0Å of the bioactive pose using the standard search protocol (and 95% success was achieved at 1.5Å using the thorough protocol). Median search time was 6.9 s for standard search and 24.7 s for the thorough protocol. Recall that the ForceGen procedure yields minimized ligand structures in all modes of operation, whereas neither OMEGA nor ConfGen do so in standard mode.

**Fig. 10:**
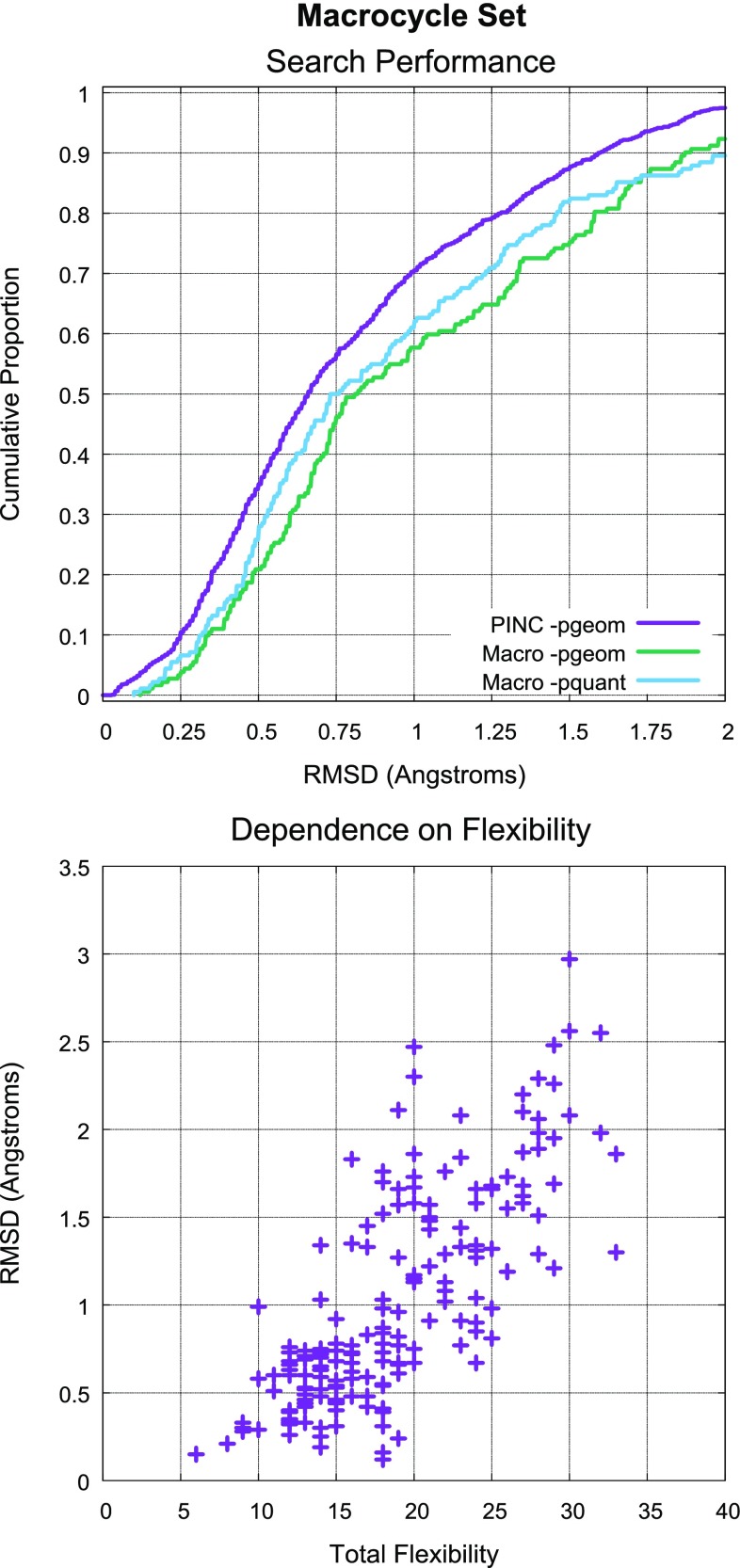
Macrocycle Set: ForceGen performance relative to the PINC Set and its relationship to molecular flexibility

With respect to the ring conformer generation ability of ForceGen, the PINC Set offers a number of complex cases, six of which are shown in Fig. 9. The CDK2 inhibitor staurosporine (top left) presents multiple challenges. It contains a fusion of a large aromatic system with a flexible bridged ring whose conformational interchange barriers are high in energy. The protein-bound form is more than 5 kcal/mol higher in energy than the lowest energy of any conformer in the ensemble. Also, the presence of multiple chiral centers complicates 3D structure generation as well as conformer elaboration. The steroid-based carbonic anhydrase inhibitor of 2GD8 binds in a low energy pose, with the close match to the X-ray structure being less than 1.5 kcal/mol above the discovered overall minimum.

**Fig. 11:**
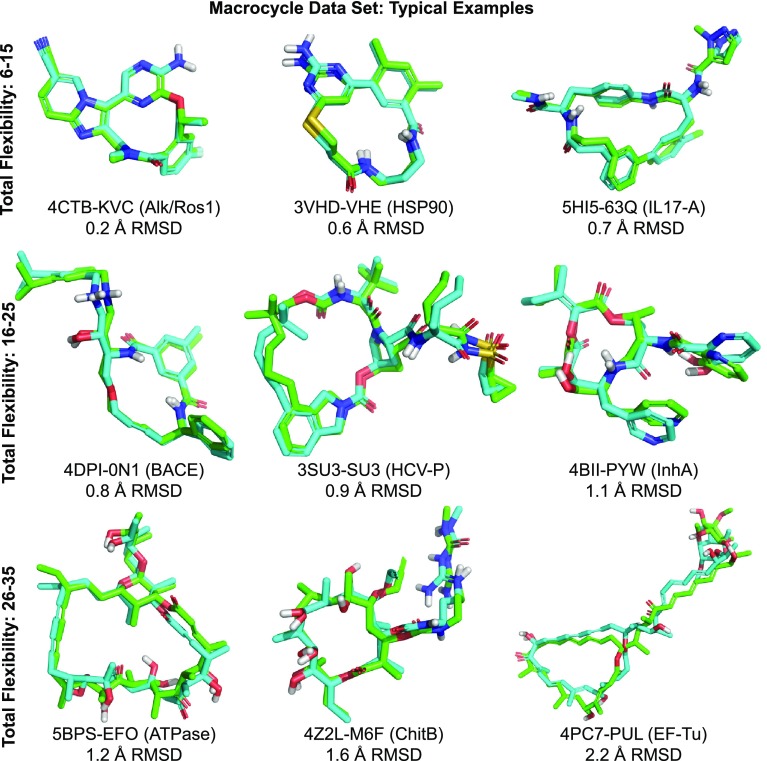
Typical conformer generation performance (standard search) for macrocycles in different classes of total flexibility

The cyclic sulfamide HIV-protease inhibitor (1AJV) is an example of the introduction of a rather novel flexible ring system that produced a surprising binding mode [31]. Recall that the ForceGen method makes use of no pre-computed templates, relying instead on its underlying force field to guide the elucidation of different conformers through a dynamic bending and twisting procedure. Dynamic ring conformer generation and all-atom minimization add somewhat to the computational cost of conformer generation relative to some template-based methods. But in the case of novel ring systems, this generality is an advantage.

The three relatively small macrocycles shown in Fig. 9 were among 22 within the PINC data set. The HSP90 ligand (3RKZ) took less than two minutes to yield 60 conformers, which included the 0.2Å best match shown. The 1NT1 thrombin ligand took less than 3 min (89 conformers), and the more flexible 3F3F BACE ligand (200 conformers) took just over 3 min. With the ForceGen approach, macrocyclic compounds need not be treated a separate class from those containing smaller ring systems. The normal non-stochastic search protocol addresses them naturally.

### Macrocycle set: large and diverse ligands

Results on the 22 macrocycles within the PINC set were encouraging: (1) the average RMSD of the best conformer generated using standard search was 1.0Å; (2) the average search time was three minutes of wall-clock time; and 86% of the cases yielded conformers within 1.5Å RMSD (and all 22 were within 2.5Å). Given this suggestive result, we curated a much larger set of bound macrocyclic ligands (182 total, process described above) limited to 60 non-hydrogen atoms or less. This cutoff was chosen so that performance on the PINC Set could be used as a point of reference.

Figure 10 depicts ForceGen performance on the Macrocycle Set relative to the PINC Set (top plot). Recall that the typical size of macrocyclic compounds was 10–15 heavy atoms larger than that seen in the PINC Set (see Fig. 4), but the distribution of freely rotatable bonds *outside* of all ring systems was very similar. So the challenge for the Macrocycle Set is, essentially, the addition of a macrocycle to the already challenging characteristics of the molecules within the PINC Set. We considered the “total flexibility” of each macrocycle to be the sum of the freely rotatable bonds plus the number of single bonds within macrocyclic rings that were not primary amides (the C-N bonds of primary amides are not twisted in the ForceGen procedure by default). There was a strong relationship between total flexibility and the RMS deviation of the closest generated conformer to the bioactive pose (see Fig. 10, bottom).

With standard search settings (200 conformer maximum), success rates for the Macrocycle Set were within roughly ten percentage points of the PINC Set. The distribution of RMS deviations was shifted rightward by approximately 0.25Å. Using the thorough search protocol (1000 conformer maximum) improved success rates by roughly 5 percentage points. The average number of conformers produced under the standard search protocol was 115, with a median search time of 183 wall-clock seconds. Under the thorough search protocol, the conformer count averaged 378, and the median search time was 370 seconds.

Figure 11 shows representative examples of ForceGen performance for three classes of macrocycles based on overall flexibility. For the least flexible set (6–15 total flexible bonds), the ForceGen approach appears to be quite accurate and also fast enough for practical use (average search time of 111 s per molecule). This group includes multiple examples of structure-enabled macrocyclic design from the last four years: an inhibitor of the anaplastic lymphoma kinase from Pfizer [32] (top left), an HSP90 inhibitor from Chugai [33] (top middle), and an IL-17A antagonist from Pfizer [34] (top right). This class of small and relatively rigid examples formed 35% of the Macrocycle Set and had mean RMSD of 0.52Å.

The moderately flexible set (16–25, middle row) formed 50% of the set, with mean RMSD of 1.07Å and mean search time of 297 s. This group included substantially more complex ligands of great pharmaceutical interest: a highly potent BACE inhibitor [35], the Merck hepatitis C therapeutic vaniprevir which targets the HCV NS3/4a protease [36, 37], and the anti-tuberculosis natural product pyridomycin which targets the InhA enoyl reductase [38].

**Fig. 12:**
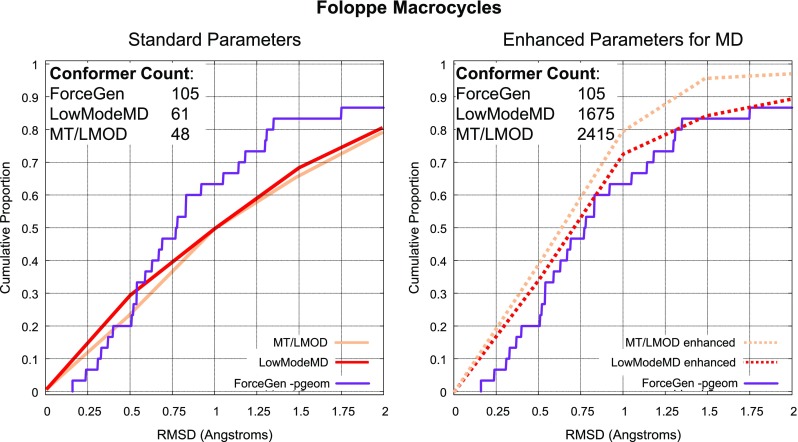
Comparison of conformer elaboration performance on the Foloppe benchmark of 30 macrocycles. Note that the plots for Low Mode MD and MT/LMOD have valid data only at the 0.5, 1.0, 1.5, and 2.0Å threshold values and that interpolated values between 0.0Å and 0.5Å are particularly biased

The two groups of compounds represented by the first two rows in Fig. 11 exemplify cutting-edge molecular design in structure-enabled lead optimization. They comprise 85% of the Macrocycle Set and appear to be tractable by the ForceGen approach, with sampling times of a few minutes each on average. Because conformer generation and docking/scoring of candidate molecules can be performed in parallel, on a 100-node cluster, nearly 30,000 compounds can be evaluated per day using this approach.

For these 154 macrocycles with total flexibility up to 25, the RMSD averaged $0.85\pm 0.51$, which matched results for the entire PINC set (RMSD average of $0.80\pm 0.53$). ForceGen produced accurate (RMSD $\le 1.5$Å) conformers for 90% of cases in this flexibility range. Note also that docking protocols often perform additional optimization of ligand poses, both using internal coordinates and Cartesian coordinates as part of the docking process [9], offering the possibility of further refinement of binding modes over the initial conformational sampling provided. We believe that ForceGen represents a significant advance and a practical means to support macrocyclic design projects with computational modeling.

The most flexible class (26 flexible bonds and up, 15% of the set) begin to challenge the ForceGen method in terms of accuracy. Average RMSD was 2.0Å, with average search times of 15 min, and just 29% achieved the 1.5Å success cutoff (39% with the thorough search protocol). Molecules within this group are still of pharmaceutical interest, but do not appear to be of as much pressing design focus as the previous examples. Whether the normal bias against large molecules as being good drug candidates holds true for macrocyclic ligands is not clear, but based on the large set of macrocycles we have curated here, it would seem that smaller molecules are receiving more focused design elaboration and structural elucidation.

Figure 11 shows three examples of the most challenging class (bottom row), which include (left to right): oligomycin A targeting the a yeast ATPase (PDB 5BPS), a macrolide chitinase inhibitor [39], and pulvomycin bound to a nucleotide-binding elongation factor [40]. Of these, only the report of the chitinase inhibitor was from a lead optimization exercise.

### Foloppe set: comparison to MD methods

Previous studies of macrocycle conformer generation have primarily made use of stochastic molecular dynamics methods [10–12], though the recently reported BRIKARD method approaches the problem using an approach from inverse kinematics [13]. Chen and Foloppe [11] introduced a set of 30 carefully curated macrocycles and reported the performance of different MD-based approaches, the best two of which were the Low Mode MD approach implemented within MOE [10] and the Mixed torsional/Low-mode (MT/LMOD) implemented within Schrödinger’s MacroModel.

**Fig. 13:**
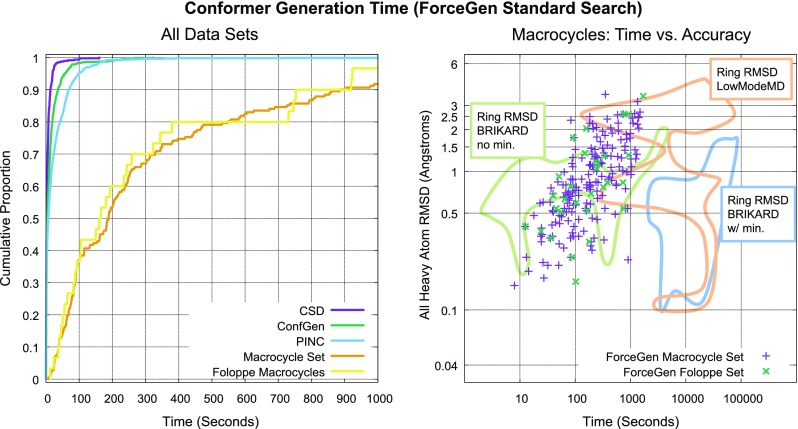
Conformer generation wall-clock timing: ForceGen performance on all four primary datasets (*left*); qualitative single-processor timing comparison to BRIKARD (both with and without minimization) and Low Mode MD methods on the BRIKARD superset of the Foloppe Set (*right*)

Figure 12 shows a comparison of the default and tuned performance of the two MD-based methods compared with ForceGen search on 30 macrocyclic compounds. Under default settings (left plot), ForceGen performed substantially better, though with just 30 data points, larger performance differences than seen here are required to establish statistical significance. By optimizing over 22 combinations of methods and parameters, a clear improvement was made, with the best optimized performance coming from Low Mode MD and MT/LMOD (right plot). ForceGen’s performance matched that of the optimized Low Mode MD method. The MT/LMOD approach appeared to show slightly better performance, though at roughly 10 percentage points, such a difference would require many more than 30 data points to reach statistical significance. Note also that to achieve the optimized performance levels, the MD-based methods produced between 10–20 times as many conformers, which has consequences for downstream calculations.

### Timing considerations

Computational cost is of great practical importance in determining whether an approach can have widespread application. Figure 13 shows the distributions of conformer search times under the standard ForceGen protocol (left plot) and a comparison of accuracy vs. search time for both the Foloppe and Macrocycle Sets along with qualitative quality/time comparisons from the report of the BRIKARD method from Coutsias et al. [13] (right plot). The timings for all methods reflect wall-clock time on single processors, though the time for Low Mode MD reflects a multi-threaded implementation. With respect to ForceGen performance, median (and average) times of small numbers of seconds are required for non-macrocyclic compounds.

Typical times for macrocyclic compounds were a few hundred seconds, with 80% requiring 10 min or less. There was a direct relationship between search time, molecular complexity, and result quality, with essentially identical patterns seen with the Foloppe and Macrocycle Sets. The BRIKARD data set included all 30 of the Foloppe macrocycles and was augmented with 37 additional compounds to increase both diversity and complexity [13]. The median time reported for the optimized Low Mode MD protocol (the protocol used in the right-hand plot from Fig. 12) was 11,000 s (roughly the middle of the orange outline in Fig. 13). The median time reported for an optimized MacroModel MD/LLMOD protocol was 37,000 s (not shown in the plot). The ForceGen approach is roughly 50–200 times faster than these competing methods.

The BRIKARD approach is quite fast in generating alternative conformations of macrocyclic systems, with timings on a single processor rivaling those of ForceGen. However, in order to achieve accuracy that compares well with ForceGen and other methods, minimization is required, which brings the BRIKARD timings into a similar region as the MD approaches using a single processor. The BRIKARD approach has a significant advantage over the MD methods. Because it is not sampling a time trajectory, it can be parallelized for searching a single molecule with a linear speedup in efficiency in relation to the number of processors. Median times using 40 processors for the BRIKARD method (with minimization) were about 400 s, which is close to the range we observed for ForceGen on a single processor.

ForceGen shares the advantage with the BRIKARD approach of not sampling a trajectory, and many stages of the algorithm can be easily parallelized across multiple computing cores. However, with single-core performance of a few minutes per molecule, most situations where computational costs would be a concern would occur when many candidate molecules require evaluation. In that case, simply spreading out different molecules across different processors provides a parallel linear speedup with no special-purpose parallel code. Making use of GPU-based acceleration might be worth pursuing, as it could enable near-real-time conformer generation for complex ligands up to and including macrocycles using ForceGen.

### Quality of ForceGen initial 3D structures

One implicit and critical aspect of the results presented here is the effect of the initial generated 3D structural model for each molecule. We do not have access to any of the popular alternative methods, so direct comparisons were not possible. However, it is important to note that the results we obtained for conformer generation depended directly on the performance of the ForceGen approach for 3D structure building. In particular, the fast screening protocol for ForceGen performs no ring elaboration. However, its performance on the ConfGen Set was competitive with the ConfGen method in its intermediate mode, despite the latter making use of template-based ring elaboration.

As we noted earlier, ForceGen 3D structure building is not as fast as template-based approaches such as CONCORD [1], CORINA [2], and OMEGA [3]. However, ForceGen’s conformational elaboration times on non-macrocycles are only slightly slower than OMEGA and are faster than ConfGen, and ForceGen’s initial structure generation takes just one-sixth of the time of conformational search on average in standard mode. Conformer elaboration is nearly always carried out in cases where 3D structures of ligands are produced, so we expect that the relatively small amount of time required for ForceGen 3D structure generation will not pose an excessive burden.

Note also that partial charge estimation is integrated into the structure generation process. Future work will address direct comparisons of initial 3D structural quality and partial charge accuracy with other methods.

## Conclusions

The results we have reported for ForceGen structure and conformer generation are comprehensive. They span small lead-like molecules from the CSD to diverse, realistic, and challenging drug-like ligands from the ConfGen and PINC data sets, which each contain significant proportions of large and flexible molecules. The results include performance on the largest set of bound macrocycles of which we are aware and also for a smaller independently curated macrocycle set. Performance analysis showed a statistically significant performance advantage over both OMEGA and ConfGen on the data sets curated for validation of each method [3, 15]. Performance on realistic drug-like ligands rivaled that seen on the CSD, with success rates reduced by just 5–10 percentage points across the 0.5–1.25 Angstrom success thresholds when using the ForceGen thorough search protocol.

Moving to the 182-compound Macrocycle Set from the challenging PINC Set, using standard search protocols, performance was reduced by roughly 10–15 percentage points when considering the full set including the most challenging cases. However, within the subset of 85% of macrocycles containing 25 or fewer total flexible bonds, performance was indistinguishable from the PINC Set. Direct comparisons with other methods on the 30 macrocycles of the Foloppe Set showed that ForceGen’s performance was superior to the default performance of both the MOE Low Mode MD method and the MacroModel MT/LMOD method. Using optimized parameters for the MD-based methods (at increased computational cost), Low Mode MD showed equivalent performance to ForceGen, and MT/LMOD showed marginally better performance with conformational ensembles 10–20 times larger than ForceGen’s. However, none of the performance differences in RMSD success rates on the Folloppe set were statistically significant due to the small data set size.

By contrast, ForceGen’s approximately 100-fold speed advantage is of clear practical significance. On a single mid-range CPU, macrocyclic compounds with the complexity level of drugs such as vaniprevir can be elaborated into ensembles of 200 conformations or less in a few minutes, with the expected RMSD of the closest conformer to the bioactive one being 0.85Å. When coupled with molecular docking, similarity, or binding affinity prediction methods, the ability to rapidly produce such high-quality ensembles should enable routine computational support for macrocyclic design projects.

The results presented here represent a remarkable demonstration of the breadth, robustness, and accuracy of the MMFF94 force field [16–21]. The ForceGen approach is geared entirely toward locating diverse minima within the energy surface dictated by the force field being used. Even with the successful application seen here, there is room for improvement of the sampling approaches under our MMFF94sf force field variant. It is possible to directly examine this question by making use of the “correct” conformation as a starting point for conformational elaboration rather than randomizing the conformations of molecules. In such a procedure, the ensemble generated will contain the nearest minimum close to the correct pose unless the combination of sampling and energy calculations drive the ensemble away. Under such a protocol, we observed that even in the simplest case of the CSD data, the ensembles were improved, with greater improvements seen on more challenging molecules.

Our future work in this area will focus on additional improvements in sampling, with relatively less effort addressing cases where there are clear issues with force field parameters. There is room for practically meaningful and statistically significant improvement, especially for complex molecules, both in terms of conformational ensemble quality as well as computational speed.
